# Fostering the international interoperability of clinical research networks to tackle undiagnosed and under-researched rare diseases

**DOI:** 10.3389/fmed.2024.1415963

**Published:** 2024-11-13

**Authors:** Galliano Zanello, Chun-Hung Chan, Samantha Parker, Daria Julkowska, David A. Pearce

**Affiliations:** ^1^Institut National de la Santé et de la Recherche Médicale, Paris, France; ^2^International Rare Diseases Research Consortium, Paris, France; ^3^Sanford Research, Sioux Falls, SD, United States; ^4^Italfarmaco S.p.A, Milan, Italy; ^5^Sanford School of Medicine, University of South Dakota, Sioux Falls, SD, United States

**Keywords:** rare diseases, undiagnosed diseases, clinical research, clinical research networks, IRDiRC

## Abstract

Clinical research is an essential component to advance diagnosis and therapeutic development. In 2022, the International Rare Diseases Research Consortium (IRDiRC) and the European Joint Programme on Rare Diseases (EJP RD) brought together key stakeholders from across the globe to discuss common themes in clinical research networks (CRNs) for rare diseases. Various topics were raised during discussions including current state of CRNs, the need for new CRNs, multi-stakeholder perspectives on value of CRNs, and ways to collaborate on a global scale. Communication and coordination between various groups, taking advantage of existing experiences, can expedite establishment and execution of complex collaborations that will be necessary for CRNs. In this perspective, we discuss opportunities and highlight key considerations for developing successful collaborative CRNs across the globe.

## Introduction

Rare diseases affect small and geographically dispersed patient populations. These often-complex disorders require multiple and interdisciplinary expert consultations to guide the implementation of the most suitable care pathway. Although individually rare, these disorders could collectively affect more than 400 million people worldwide making them a real challenge for healthcare systems and a global public health priority (1, 2). Despite the tremendous efforts of the rare disease community to accelerate research and care, most rare disease patients still lack a timely diagnosis and approved orphan therapies for their condition. Clinical research networks (CRNs) for rare diseases have been developed at the national or continental level and their scope may differ depending on field of research and geographical coverage (3). For example, European Reference Networks (ERNs) have been set up, in the European Union, to create reference points and compensate the problem of dispersed small patient populations, and support patients and multi-disciplinary teams to gain better understanding on specific rare disorders (4). CRNs have been established in the United Kingdom (UK), funded by the UK Health Department, to provide infrastructure to support high quality clinical research studies for the benefit of patients (5). In the United States, the Rare Diseases Clinical Research Network (RDCRN) funded by the National Institutes of health (NIH), fosters collaborative research among scientists to better understand how particular rare diseases progress and to develop improved approaches for diagnosis and treatment (6). In Japan, the Initiative on Rare and Undiagnosed Diseases (IRUD), launched by the Japan Medical and Research Development Agency (AMED), is a clinical research program aiming to support the diagnosis of patients with undiagnosed disease via data sharing and to promote research into pathology that may lead to the development of new therapies (7). However, global collaborative efforts to connect CRNs are often lacking thus slowing their full potential in accelerating diagnosis and therapy development. Notably, these joint efforts could accelerate the collection and standardization of data on undiagnosed and under-researched rare diseases often characterized by very low prevalence and high complexity, and subsequently facilitate the implementation of drug development programs addressing better-defined patients` needs and expectations.

The International Rare Diseases Research Consortium (IRDiRC) is a global collaborative initiative, including public and non-for-profit funders, regulatory bodies, academics, patient umbrella groups and biopharmaceutical and diagnostic companies, aiming to tackle rare diseases through research and accelerate diagnosis and therapy development for rare diseases (8, 9). IRDiRC launched a Task Force in 2020 to analyze the structure and attributes of CRNs for rare diseases, including the key elements conducive to collaboration, and to identify the barriers and needs preventing their interoperability (10). The hurdles identified by the Task force were grouped into five categories addressing: funding limitation; lack of harmonization in regulatory and contracting process; need for common tools and data standards; need for a governance framework and coordination structure; and lack of awareness and robust interactions between networks. The task force also revealed that biopharmaceutical companies are poorly integrated into the CRNs (3).

Tackling these issues will require joint efforts from multiple stakeholders namely—patients, clinicians, researchers, industry, regulators, funders, policy makers—located in different geographical areas and working in different jurisdictions. Understanding the specificity of each network, defining common goals and the means to achieve them will be essential since CRNs for rare diseases do not all have the same mandate. An agreement on a global collaborative framework for CRNs could be a major steppingstone to the creation of a long-term roadmap supported by concrete actions and leveraging the experience of the existing networks.

Since IRDiRC member organizations represent all continents and due to its nature and commitments, IRDiRC can stimulate stakeholders’ interactions and support the development of a roadmap for the international collaboration and interoperability of CRNs for rare diseases. Here, we present the perspective of IRDiRC on the topic.

## Know your neighbor—the importance of mapping and networking of clinical research networks for rare diseases

**Figure 1:**
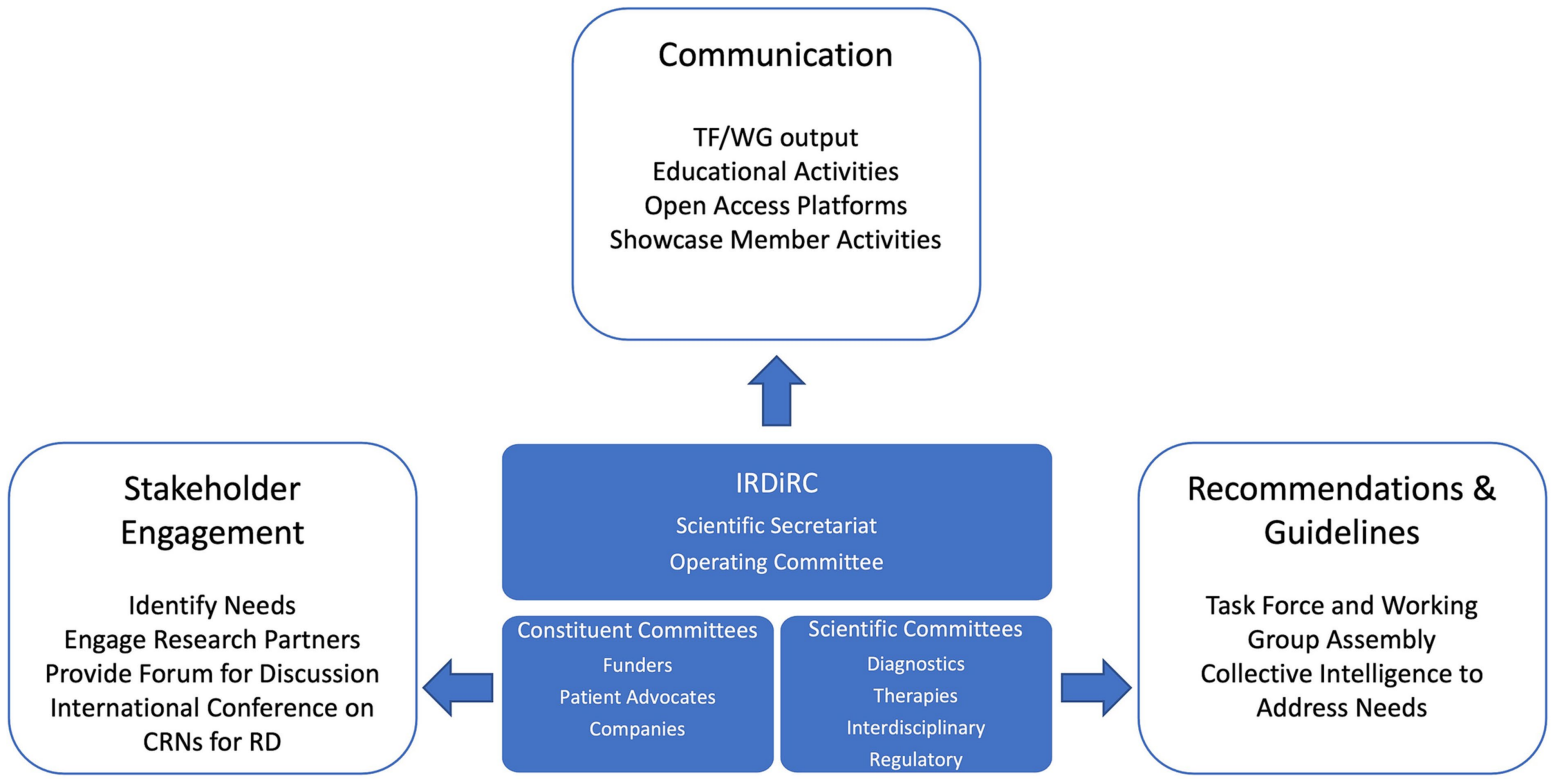
Mechanisms by which the International Rare Diseases Research Consortium (IRDiRC) can support the development and the collaboration of clinical research networks (CRNs) for rare diseases (RD) across the globe. By sharing collective intelligence in a non-competitive space, IRDiRC can rely on its constituent and scientific committees to foster worldwide multi-stakeholder engagement and can serve as a forum to discuss the development, the collaboration and the interoperability of CRNs for rare diseases. Continued monitoring and interactions with the global rare disease community connect IRDiRC with initiatives and organizations working toward the development and the interoperability of CRNs across the globe. Task Forces (TF) and Working Groups (WG) are the instruments used by IRDiRC to provide recommendations and guidelines that will help the community in developing collaborative models and tools to support CRNs interoperability. IRDiRC can rely on its network to communicate and disseminate the relevant material and information to advancing collaboration.

CRNs for rare diseases present a wide diversity of mandates and activities from improving the diagnosis of rare disease patients to accelerating clinical trials, care management and therapy development (3). To define the framework of collaboration that can form the basis for CRNs’ international interoperability and to implement joint actions, it is essential for CRNs to be aware of other existing networks, their structure, goals, and capacities. Such initial interactions can be facilitated by the organization of regular events dedicated to cross-CRN exchange aiming to share knowledge and identify collaborative pathways. The first international conference on CRNs for rare diseases was co-organized by IRDiRC and the European Joint Programme on Rare Diseases (EJP RD) (11) in December 2022 to gather experts from different continents and increase mutual knowledge on CRNs structure, activities and identify routes to stimulate cooperation and interoperability of these networks (12). IRDiRC, in partnership with the newly launched European Rare Diseases Research Alliance (ERDERA) (13, 14), is committed to support the organization of future conferences offering a forum to discuss the implementation of concrete actions and to stimulate the engagement of additional experts and organizations. As a global collaborative initiative of rare disease stakeholders, IRDiRC is well positioned to facilitate the international collaboration and interoperability of CRNs through its organization and activities as shown in Figure 1.

## The international value of data

Undiagnosed and under-researched rare diseases are often characterized by very low prevalence and highly complex genotype–phenotype association. The limited knowledge on the etiology and the progression of these diseases, and the difficulty to capture regulatory-grade data for the initiation of drug development programs leave too many patients and families without consensus recommendations and approved medicinal products for managing and treating these diseases. Due to their complexity and extreme rarity, patients are—when possible—referred to CRNs to gain better disease understanding through consultation with multi-disciplinary teams. In this context, the multiplication of data sources using different nomenclature, the absence of concerted agreement on data collection, outcomes and endpoints selection, and the lack of interoperability of the systems, including the use of common consent forms, the standardization of data and the possibility to share data across networks, are major hurdles slowing down clinical research and limiting the opportunity for rare disease patients to gain access to clinical trials.

In the context where every data element is precious and can contribute to more accurate and faster diagnosis and treatment, the international dimension that CRNs can bring is of considerable importance. The collaboration between networks could facilitate and prioritize the harmonization and set-up of registries for under-researched rare diseases and accelerate the collection and validation of regulatory-grade data (e.g., biomarkers, clinical outcome assessments and endpoints) to expedite clinical trial readiness and inform drug development programs. Through concerted actions, CRNs for rare diseases could increase their legitimacy as expert partners for regulatory agencies to define the scientific context for data collection and use, including real world data to support multinational clinical studies and complex designs for clinical trials (15). In this context, concertation between regulatory agencies to accelerate the harmonization of the processes governing the capture of data, including real-world data and real-world evidence, and the conduct of clinical studies could greatly facilitate the implementation of drug development programs and the measure of medicine effectiveness (16). Furthermore, resources such as the EJP RD Virtual Platform (17, 18) and the Rare Disease Cures Accelerator-Data and Analytics Platform (19) that adhere to and promote FAIR (Findable, Accessible, Interoperable, Reusable) principles, and which have been designed and created to accelerate discovery and access to patient-level data, could greatly support CRNs in achieving their interoperability, data collection and access processes while preserving privacy of patients.

## Connecting the dots through patient empowerment and partnerships

Patient empowerment and partnerships are cornerstones for CRNs as patient groups have the power to connect clinical teams around the world and guide them in respect to patients’ needs and expectations. Moreover, internationally linked CRNs can be entry points and enhancers for under-researched rare diseases where no patient organizations exist and the need to connect different stakeholders is decisive. To support such engagement, funders may consider launching funding opportunities to support patient groups proposing research projects on under-researched rare diseases, connecting multiple clinical research teams linked to different CRNs, while building a multi-stakeholder community encompassing industry participation. Importantly, IRDiRC’s Funders Constituent Committee or Board of Funders of EJP RD (and of the newly launched ERDERA) can be game changers in this area.

## Rare diseases without frontiers

Analysis of existing CRNs demonstrated that depending on the regional or national priorities, and political context of their creation, CRNs may be more research or healthcare oriented (3). However, in the rare disease space clinical care and research are strongly integrated. In some cases, research and clinical care are considered a continuum, as clinical care and observations are essential to inform research which in turn will inform healthcare decisions. The discussions of the *International Conference on CRNs for Rare Diseases* (12) made clear that many regions or countries are still exploring how to create, or are preparing to launch, a CRN in their geographical area.

Following these lines, it is expected that the collaboration between CRNs for rare diseases should contribute to reduce inequities between geographies and support IRDiRC’s vision to leave no one behind by promoting education and training. To reach this vision, sharing of knowledge among RD stakeholders, including expert health care providers involved in the joint assessment of the most complex patient cases, maximized data collection efforts and support in the initiation of clinical research studies in multiple networks should happen irrespectively of CRNs geography (20).

An additional consideration for the success of CRNs in conducting research is their interactions and collaboration with the biopharmaceutical industry. CRNs have a potential to improve the drug development process by delivering multiple activities related to clinical trials including the nonclinical proof-of-concept research, designing studies with a patient-centric approach, leading patient registries and natural history studies, and facilitating patient recruitment into clinical trials. For this to become a reality CRNs need to build a pathway for industry to be considered as a research partner.

## Conclusion

The creation of CRNs for rare diseases at the national and continental level has been shown to be essential to gain better understanding of specific rare diseases and accelerate clinical research, diagnosis and therapy development. However, for most of the under-researched rare diseases, the size of the patient population at the national or even continental level is not sufficient to generate critical mass of knowledge on the diseases and attract the interest of drug developers. The collaboration and the interoperability of CRNs for rare diseases should aim to overcome these hurdles. By connecting on a global scale, developing joint roadmaps for improved collection and access to standardized data, sharing expert knowledge, and benefiting from collective input and experience of patients irrespectively of their geography, socio-economic and cultural status, the CRNs can demonstrate the unprecedent power of cooperation for the benefit of all people living with a rare disease.

## Data Availability

The original contributions presented in the study are included in the article/supplementary material, further inquiries can be directed to the corresponding author.
